# Chemoimmunotherapy Combined With Antiangiogenic Therapy in Advanced Triple‐Negative Breast Cancer: Real‐World Outcomes From a Retrospective Cohort Study

**DOI:** 10.1002/mco2.70839

**Published:** 2026-07-05

**Authors:** Yudong Li, Jinna Lin, Mengdi Zhu, Zijie Cai, Liang Jin, Shunying Li, Qianfeng Shi, Hongna Lai, Chang Gong, Qiang Liu

**Affiliations:** ^1^ Guangdong Provincial Key Laboratory of Malignant Tumor Epigenetics and Gene Regulation, Sun Yat‐Sen Memorial Hospital Sun Yat‐Sen University Guangzhou China; ^2^ Breast Tumor Center, Sun Yat‐Sen Memorial Hospital Sun Yat‐Sen University Guangzhou China

**Keywords:** advanced breast cancer, antiangiogenic therapy, immunotherapy, immune checkpoint inhibitor, triple‐negative breast cancer

## Abstract

Triple‐negative breast cancer (TNBC) has a poor prognosis and limited treatment options. Although programmed death protein 1 (PD‐1) inhibitor‐based chemotherapy and its combination with antiangiogenic agents are increasingly used clinically, comparative effectiveness data are lacking. This real‐world study compared chemoimmunotherapy (IC) versus chemoimmunotherapy plus antiangiogenic therapy (ICA) in advanced TNBC. Among 218 eligible patients, the ICA group (*n* = 119) showed a significantly higher disease control rate (DCR) than the IC group (*n* = 99) (85.7 vs. 73.7%, *p *= 0.009). Notable numerical improvements were observed in high‐risk subgroups: in patients with ≥3 prior lines (*n* = 56), ICA improved DCR by 26.0% (81.6 vs. 55.6%, *p *= 0.039) and prolonged PFS by 2.9 months (5.3 vs. 2.4 months, *p *= 0.031). In those with brain metastases (*n* = 24), ICA increased DCR by 50.0% (83.3 vs. 33.3%, *p *= 0.017, posthoc power = 55.4%), with extended progression‐free survival (PFS) (+4.0 months) and overall survival (OS) (+12.6 months). Early recurrence patients (disease‐free interval (DFI) < 12 months, *n* = 54) showed a 28.6% higher DCR with ICA (78.6 vs. 50.0%, *p *= 0.024). This study highlights the potential of adding antiangiogenic therapy to chemoimmunotherapy, particularly for high‐risk or refractory advanced TNBC patients, suggesting a strategy that may overcome programmed death ligand 1 (PD‐L1)‐related resistance and warrants prospective validation.

## Graphical abstract

This retrospective real‐world study compared the efficacy of IC versus ICA in 218 patients with advanced TNBC. ICA showed a higher DCR (85.7 vs. 73.7%, *p *= 0.009) and conferred benefits on refractory subgroups (+26% DCR in ≥3 prior lines, +50% in brain metastases, +28.6% in early recurrence). Notably, PD‐L1 expression failed to predict differential efficacy. These findings highlighted ICA's potential to overcome resistance and warranted randomized trials to establish ICA as a standard for high‐risk TNBC.

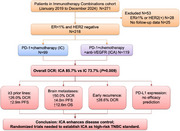

## 1 Introduction

Triple‐negative breast cancer (TNBC), accounting for approximately 15–20% of all breast cancer cases, is characterized by poor differentiation, high invasiveness, a marked propensity for local and distant metastasis, and consequently, dismal prognosis with a high recurrence risk [1]. For patients with metastatic TNBC, the median overall survival (OS) remains dismally short, spanning merely 12–18 months [2], underscoring the urgent need for more effective therapeutic strategies.

In recent years, the advent of immune checkpoint inhibitors (ICIs), particularly PD‐1 inhibitors, has revolutionized cancer treatment [3, 4]. However, TNBC demonstrates limited response to ICI monotherapy [3, 5, 6], despite exhibiting elevated programmed cell death 1 ligand 1 (PD‐L1) expression [3], increased tumor‐infiltrating lymphocytes [7], and higher tumor mutational burden compared with other breast cancer subtypes [8]. Pivotal trials such as KEYNOTE‐012 [9], KEYNOTE‐028 [10], KEYNOTE‐086 [11, 12], and JAVELIN/Avelumab [13, 14] consistently demonstrate subtherapeutic efficacy of PD‐1/PD‐L1 inhibitor monotherapy in advanced TNBC. This has led to the exploration of combination strategies.

The Phase III IMpassion130 [15] trial was practice‐changing, demonstrating that adding atezolizumab (an anti‐PD‐L1 antibody) to nab‐paclitaxel significantly prolonged both median progression‐free survival (PFS) and OS in patients with PD‐L1‐positive advanced TNBC, leading to its regulatory approval. Similarly, KEYNOTE‐355 showed pembrolizumab plus chemotherapy significantly improved PFS and OS compared with chemotherapy alone in previously untreated metastatic TNBC expressing PD‐L1 with a combined positive score (CPS) ≥ 10, validating PD‐1/PD‐L1 inhibition in biomarker‐selected advanced TNBC [16]. However, subsequent trials revealed critical heterogeneity in outcomes. IMpassion131 trial failed to show a significant PFS improvement for atezolizumab plus paclitaxel versus paclitaxel alone, with an exploratory trend toward shorter OS, contrasting starkly to the positive outcomes observed in the IMpassion130 [17]. Furthermore, IMpassion132 [18], focused on early‐relapsing (recurrence <12 months postcurative therapy) TNBC, found no benefit from adding atezolizumab to platinum‐based chemotherapy. These divergent findings underscore the heterogeneity of treatment response and emphasize the necessity for optimized patient selection and novel combination strategies.

Vascular endothelial growth factor (VEGF) overexpression in 30–60% of TNBC drives angiogenesis, creating an immunosuppressive microenvironment characterized by abnormal vasculature and restricted immune cell infiltration. Antiangiogenic therapies, such as VEGFR inhibitors, normalize tumor vasculature and enhance immune cell trafficking while upregulating PD‐L1 expression, thereby potentiating ICIs [19, 20, 21]. Our preclinical studies revealed that low‐dose antiangiogenics reprogram the immunosuppressive tumor microenvironment, significantly improving anti‐PD‐1/PD‐L1 efficacy [22]. Clinically, camrelizumab (anti‐PD‐1) combined with apatinib (VEGFR2 inhibitor) demonstrated a 43.3% objective response rate (ORR) in advanced TNBC regardless of PD‐L1 status or treatment line, outperforming historical monotherapy ORRs (5.2–18.5%) [23]. Similarly, a triplet regimen of atezolizumab, paclitaxel, and bevacizumab yielded median PFS of 11.0 months and median OS of 27.4 months in first‐line metastatic TNBC [24]. Notably, camrelizumab/apatinib/eribulin achieved 37.0% ORR and 87.0% disease control rate (DCR) in heavily pretreated patients [25]. These findings validate the mechanistic synergy and clinical potential of antiangiogenics–ICI combinations to overcome immune evasion in TNBC.

Despite these advances, a critical gap remains in the direct comparison of standard chemoimmunotherapy (IC) versus chemoimmunotherapy augmented with an antiangiogenic agent (ICA) in the real‐world management of advanced TNBC. Such data are essential to inform clinical decision‐making, especially for patients with high‐risk features or refractory disease. This study aimed to address this gap through a comprehensive retrospective analysis, evaluating and comparing the efficacy and potential subgroup benefits of IC versus ICA regimens in a real‐world cohort of advanced TNBC patients.

## 2 Results

### 2.1 Baseline Characteristics and Therapeutic Outcomes in TNBC (ER < 1%)

#### 2.1.1 Comparative Efficacy of Immunotherapy Combinations

**FIGURE 1:**
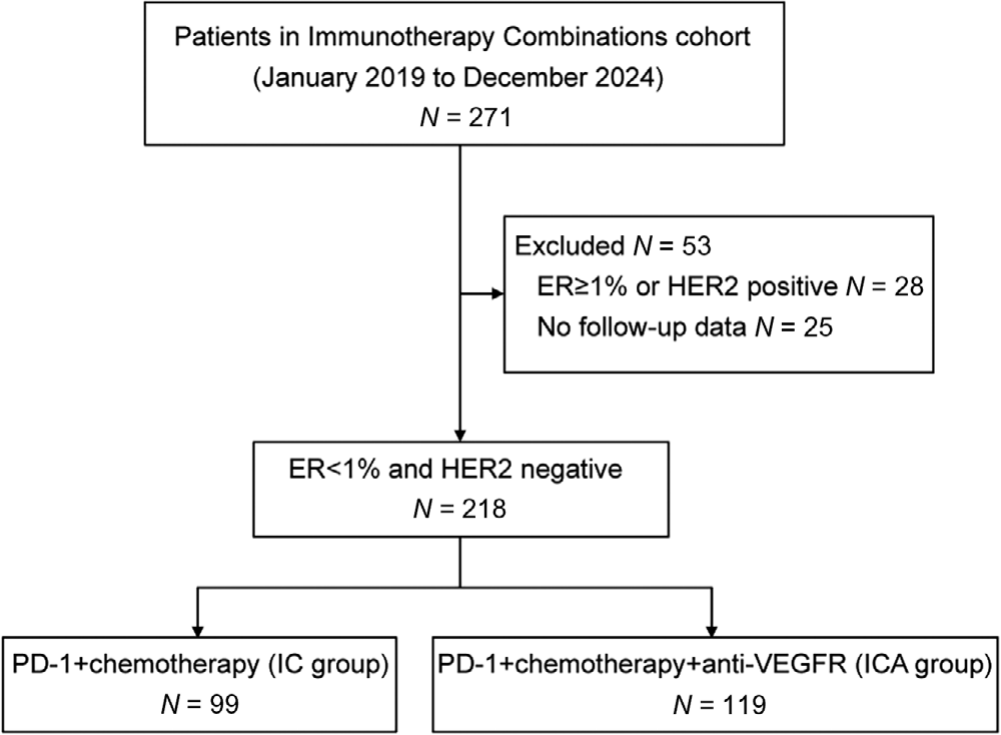
Study flow chart. ER, estrogen receptor; HER2, human epidermal growth factor receptor 2; PD‐1, PD‐1 inhibitors.

This retrospective study initially screened 271 patients with advanced TNBC treated with PD‐1 inhibitor at the Breast Tumor Center, Sun Yat‐sen Memorial Hospital between 2019 and 2024. After applying the inclusion and exclusion criteria, 218 patients with ER‐negative (<1%) were included for efficacy assessment [median follow‐up, 19.3 months (95% confidence interval [CI] 15.7–22.9 months)] (Figure 1). Patients were stratified into treatment groups: PD‐1 inhibitor plus chemotherapy (IC group, *n* = 99, 45.4%) and PD‐1 inhibitor plus chemotherapy and anti‐vascular endothelial growth factor receptor (VEGFR) therapy (ICA group, *n* = 119, 54.6%). The results showed no significant differences in baseline age, histological type, or proportion of visceral metastasis between the two groups (*p* > 0.05). However, the ICA group had significantly higher proportion of late‐line therapy recipients (≥3 prior regimens, 31.9 vs. 18.2%, *p *= 0.021) and brain metastasis (15.1 vs. 6.1%, *p* = 0.033), indicating greater baseline disease burden in the ICA group (Table 1).

**TABLE 1:** Baseline disease characteristics of TNBC patients.

	Total *N* = 218	IC group *N* = 99	ICA group *N* = 119	*p* Value
**Age (years)**				
Mean ± SD	44.6 ± 9.4	45.1 ± 10.0	44.2 ± 8.9	0.486
<40	69 (31.7)	29 (29.3)	40 (33.6)	0.495
≥40	149 (68.3)	70 (70.7)	79 (66.4)	
**Histological type**				
Ductal	196 (89.9)	89 (89.9)	107 (89.9)	0.997
Other	22 (10.1)	10(10.1)	12(10.1)	
**Therapy line**				
Line 1–2	162 (74.3)	81 (81.8)	81 (68.1)	0.021
Line ≥ 3	56 (25.7)	18 (18.2)	38 (31.9)	
**Visceral metastasis**				
Yes	105 (48.2)	47 (47.5)	58 (48.7)	0.852
No	113 (51.8)	52 (52.5)	61 (51.3)	
**Brain metastasis**				
Yes	24 (11.0)	6 (6.1)	18 (15.1)	0.033
No	194 (89.0)	93 (93.9)	101 (84.9)	
**Disease‐free interval (DFI)**				
De novo Stage IV	43 (19.7)	25 (25.3)	18 (15.1)	0.101
<12 month	54 (24.8)	26 (26.3)	28 (23.5)	
≥12 month	121 (55.5)	48 (48.5)	73 (61.3)	
**Best overall response**				
CR (%)	18 (8.3)	7 (7.1)	11 (9.2)	0.151
PR (%)	60 (27.5)	27 (27.3)	33 (27.7)	
SD (%)	97 (44.5)	39 (39.4)	58 (48.7)	
PD (%)	43 (19.7)	26 (26.3)	17 (14.3)	
ORR (%)	78 (35.8)	34 (34.3)	44 (37.0)	0.432
95% CI	29.4–42.2	24.8–43.9	28.2–45.8	
DCR (%)	175 (80.3)	73 (73.7)	102 (85.7)	0.009
95% CI	75.0–85.6	64.9–82.6	79.3–92.1	
PFS (median)	7.0m	7.1m	6.9m	0.400
95% CI	6.1–7.9	4.4–9.8	5.8–8.0	
≥6 month (%)	104 (59.2)	44 (61.7)	60 (57.5)	
≥1 year (%)	42 (32.7)	16 (34.7)	26 (31.3)	
≥2 year (%)	15 (16.2)	7 (20.2)	8 (13.6)	
OS (median)	19.8m	19.4m	20.1m	0.300
95% CI	15.7–23.9	14.0–24.8	14.6–25.6	
≥1 year (%)	100 (72.0)	34 (69.6)	66 (73.4)	
≥2 year (%)	38 (41.0)	13 (37.2)	25 (43.5)	
≥3 year (%)	14 (25.0)	4 (21.5)	10 (27.2)	

Abbreviations: CI, confidence interval; CR, complete response; DCR, disease control rate; IC group, PD‐1 inhibitors combined with chemotherapy; ICA group, PD‐1 inhibitors combined with chemotherapy plus anti‐VEGFR agents; ORR, objective response rate; OS, overall survival; PD, progressive disease; PFS, progression‐free survival; PR, partial response; SD, stable disease; SD, standard deviation.

Although there were no significant differences in ORR (34.3 vs. 37.0%, adjust odds ratio [aOR] 1.26, 95% CI 0.71–2.24, *p *= 0.432), median PFS (7.1 vs. 6.9 months, adjusted hazard ratio [aHR] 0.86, 95% CI 0.60–1.22, *p *= 0.400), and median OS (19.4 vs. 20.1 months, aHR 0.80, 95% CI 0.53–1.22, *p *= 0.300), the ICA group achieved a statistically higher DCR (85.7 vs. 73.7%, aOR 2.61, 95% CI 1.27–5.38, *p *= 0.009). Notably, numerical improvements were observed in the 1‐year OS rate (73.4 vs. 69.6%), 2‐year OS rate (43.5 vs. 37.2%), and 3‐year OS rate (27.2 vs. 21.5%) for the ICA group (Table 1 and Figure S1).

#### 2.1.2 Treatment‐Line Dependent Therapeutic Efficacy

Further analysis showed that patients receiving first‐line or second‐line immunotherapy (*n* = 162) demonstrated a higher ORR (39.5 vs. 25.0%, aOR 0.49, 95% CI 0.24–0.97, *p *= 0.041, a higher DCR (82.7 vs. 73.2%, aOR 0.46, 95% CI 0.22–0.98, *p *= 0.044), significantly prolonged median PFS (8.4 vs. 4.6 months, Δ+3.8 months, aHR 2.21, 95% CI 1.52–3.22, *p *< 0.001), and significantly improved median OS (20.7 vs. 15.6 months, Δ+5.1 months, aHR 1.79, 95% CI 1.13–2.82, *p *= 0.013) compared with patients receiving ≥third‐line immunotherapy (*n* = 56). Additionally, consistently superior long‐term PFS/OS rates were observed in early‐line cohorts (Figure S2). Collectively, these data suggest that upfront immunotherapy may be associated with a trend toward improved survival in patients with advanced TNBC.

#### 2.1.3 Line‐Specific Synergy of Anti‐VEGFR Combination

**FIGURE 2:**
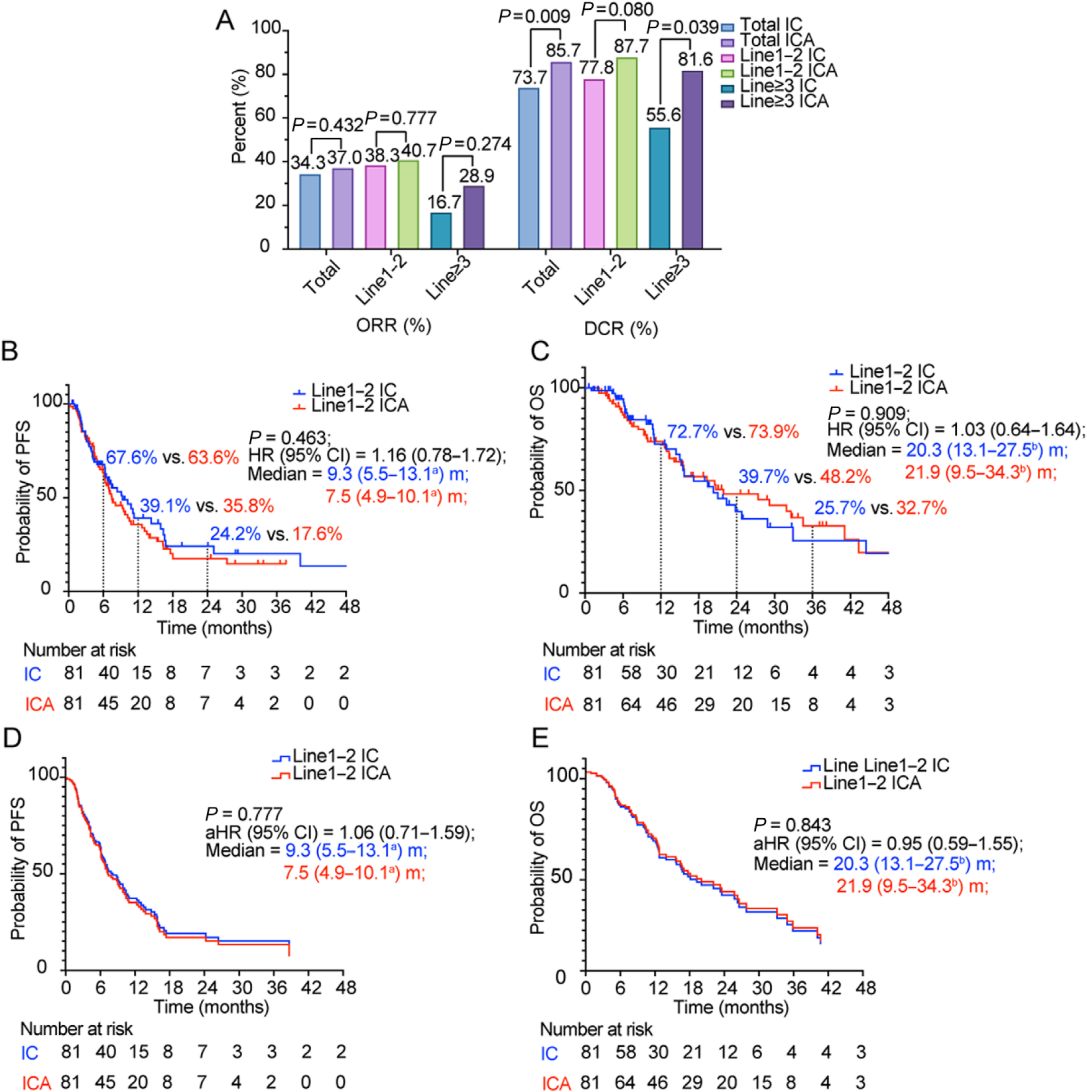
Efficacy of immunization combination regimens under different treatment lines in advanced TNBC. (A) ORR and DCR for IC group and ICA group by treatment line. (B) and (C) PFS and OS in line 1–2 patients receiving IC or ICA therapy by Kaplan–Meier method with log‐rank test; (D) and (E) PFS and OS in line 1–2 patients receiving IC or ICA therapy by Cox proportional hazards models. IC, PD‐1 inhibitors combined with chemotherapy; ICA, PD‐1 inhibitors combined with chemotherapy plus anti‐VEGFR agents; ORR, objective response rate; DCR, disease control rate; PFS, progression‐free survival; OS, overall survival; HR, hazard ratio; aHR, adjust hazard ratio; CI, confidence interval; *Note*: ^a^95% CI of median PFS; ^b^95% CI of median OS.

**FIGURE 3:**
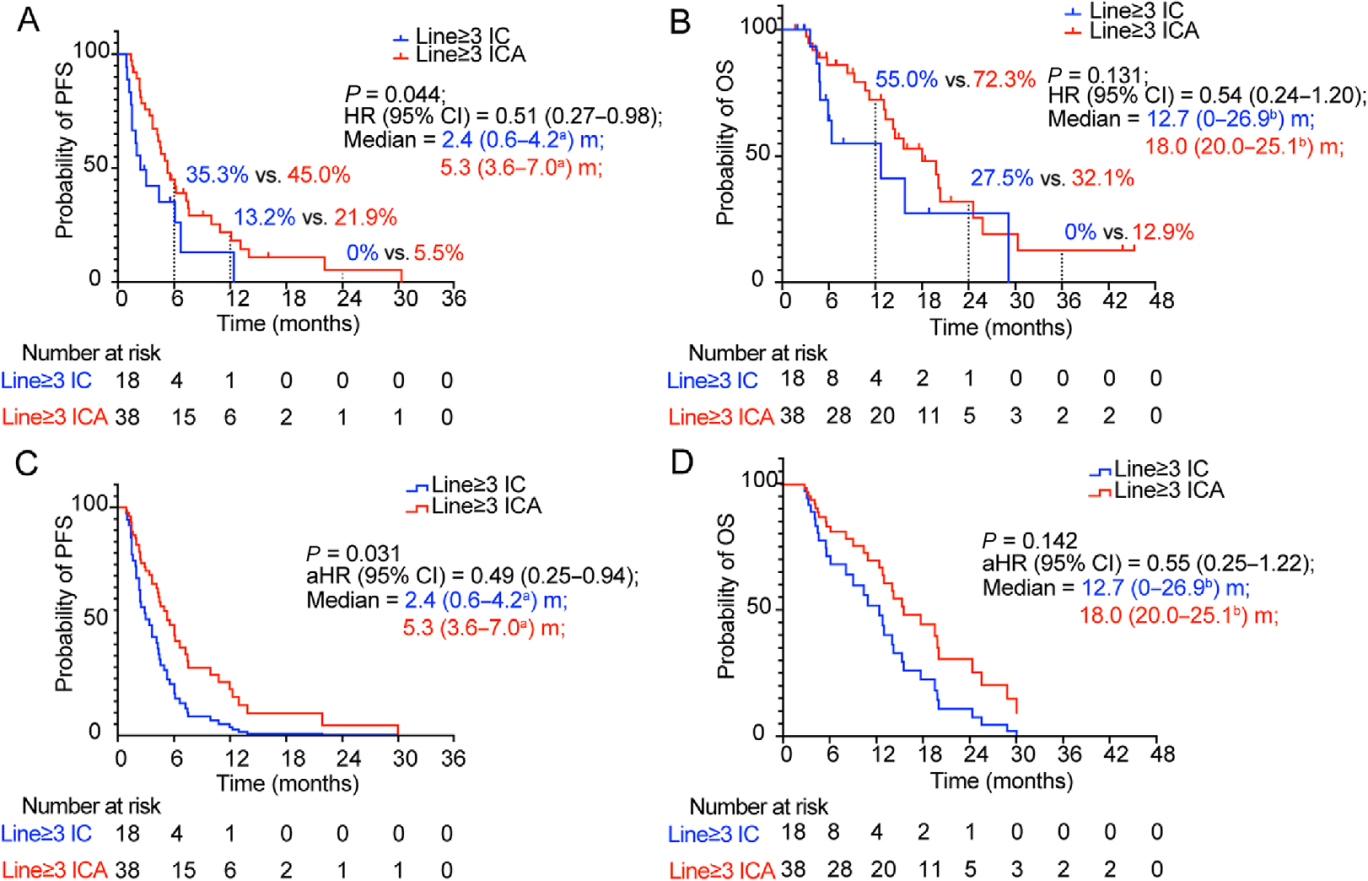
Efficacy of immunization combination regimens in line ≥ 3 patients receiving IC or ICA therapy. (A) and (B) PFS and OS in line ≥ 3 patients receiving IC or ICA therapy by Kaplan–Meier method with log‐rank test; (C) and (D) PFS and OS in line ≥ 3 patients receiving IC or ICA therapy by Cox proportional hazards models. PFS, progression‐free survival; OS, overall survival; HR, hazard ratio; aHR, adjust hazard ratio; CI, confidence interval; *Note*: ^a^95% CI of median PFS; ^b^95% CI of median OS.

Analysis of different immunotherapy combinations across treatment lines demonstrated that among patients receiving ≥third‐line therapy (*n* = 56), the ICA group showed a higher DCR (81.6 vs. 55.6%, aOR 3.81, 95% CI 1.07–13.52, *p *= 0.039), significantly prolonged median PFS by 2.9 months (5.3 vs. 2.4 months, aHR 0.49, 95% CI 0.25–0.94, *p *= 0.031), and extended median OS by 5.3 months (18.0 vs. 12.7 months, aHR 0.55, 95% CI 0.25–1.22, *p *= 0.142), although the OS improvement did not reach statistical significance (Figures 2 and 3). The 1‐year OS rate was also higher in the ICA group (72.3 vs. 55.0%) compared with the IC group (Figure 3). In contrast, no significant differences in ORR, median PFS, or median OS were observed between the IC group and ICA group in the first‐/second‐line cohorts (Figure 2).

### 2.2 Efficacy Analysis Based on PD‐L1 CPS

#### 2.2.1 Association Between PD‐L1 CPS and Efficacy

Among 123 patients with evaluable PD‐L1 CPS, we detected no significant differences in ORR, DCR, median PFS, or median OS between CPS ≥ 10 (*n* = 55) and CPS < 10 (*n* = 68) groups (all *p *> 0.05) (Table 2). These data suggest that in this real‐world cohort receiving combination therapy, PD‐L1 CPS status (using a ≥10 cutoff) may not robustly discriminate outcomes, highlighting the complexity of biomarker interpretation in the context of combination regimens. More systematic and comprehensive models for predicting immunotherapy efficacy remain to be explored.

**TABLE 2:** PD‐L1 CPS status and efficacy outcomes in patients.

	Total	CPS		*p* Value
		≥10	<10	
*N* (%)	123	55 (44.7)	68 (55.3)	
**Best overall response**				
CR (%)	7 (5.7)	5 (9.1)	2 (2.9)	0.172
PR (%)	36 (29.3)	16 (29.1)	20 (29.4)	
SD (%)	53 (43.1)	19 (34.5)	34 (50.0)	
PD (%)	27 (22.0)	15 (27.3)	12 (17.6)	
ORR (%)	43 (35.0)	21 (38.2)	22 (32.4)	0.500
95% CI	26.4–43.5	24.9–51.4	20.9–43.8	
DCR (%)	96 (78.0)	40 (72.7)	56 (82.4)	0.200
95% CI	70.6–85.5	60.6–84.9	73.1–91.6	
**Combine drug**				
IC group	56 (45.5)	28 (50.9)	28 (41.2)	0.281
ICA group	67 (54.5)	27 (49.1)	40 (58.8)	
PFS (median)	6.7m	7.1m	6.7m	0.716
95% CI	5.5–7.9	5.5–8.7	3.5–9.9	
≥6m	55 (57.1)	25 (45.5)	30 (44.1)	
≥1y	26 (38.3)	11 (20.0)	15 (22.1)	
≥2y	12 (23.7)	6 (10.9)	6 (8.8)	
OS (median)	19.3m	19.8m	15.6m	0.582
95% CI	15.1–23.5	16.2–23.4	8.1–23.1	
≥1y	52 (68.6)	26 (47.3)	26 (38.2)	
≥2y	23 (40.4)	10 (18.2)	13 (19.1)	
≥3y	10 (29.8)	4 (7.3)	6 (8.8)	

Abbreviations: CI, confidence interval; CR, complete response; DCR, disease control rate; IC group, PD‐1 inhibitors combined with chemotherapy; ICA group, PD‐1 inhibitors combined with chemotherapy plus anti‐VEGFR agents; ORR, objective response rate; OS, overall survival; PD, progressive disease; PFS, Progression‐free survival; PR, partial response; SD, stable disease.

#### 2.2.2 Efficacy of Combination Strategies by CPS Subgroup

Further analysis of combination strategies stratified by CPS revealed that adding antiangiogenic agents did not significantly improve median PFS or OS in either CPS ≥ 10 or CPS < 10 cohorts. Among patients receiving IC alone (IC group), those with CPS <10 had a lower ORR than those with CPS ≥ 10 (25.0 vs. 39.3%). With the addition of antiangiogenic therapy (ICA group), the ORR in the CPS < 10 subgroup increased to 37.5%, reaching a level comparable to that in the CPS ≥ 10 subgroup (37.0%), whereas patients with CPS ≥ 10 derived no additional numerical ORR benefit from this combination. In an exploratory analysis of 18 patients with CPS < 1 receiving ICIs plus antiangiogenic therapy (ICA group), the ORR reached 38.9%. Notably, this was achieved despite 55.6% (10 out of 18) of patients having received ≥3 prior lines of therapy and was comparable to the 37.0% benchmark observed in the CPS ≥ 10 population. Additionally, this combination was associated with numerically improved long‐term survival rates (≥2‐year and ≥3‐year OS) in this CPS < 1 population (Table S1). Given the very small sample size, these findings are descriptive only.

### 2.3 Exploratory Analyses

#### 2.3.1 Enhanced Efficacy in Brain Metastasis Cohort

**FIGURE 4:**
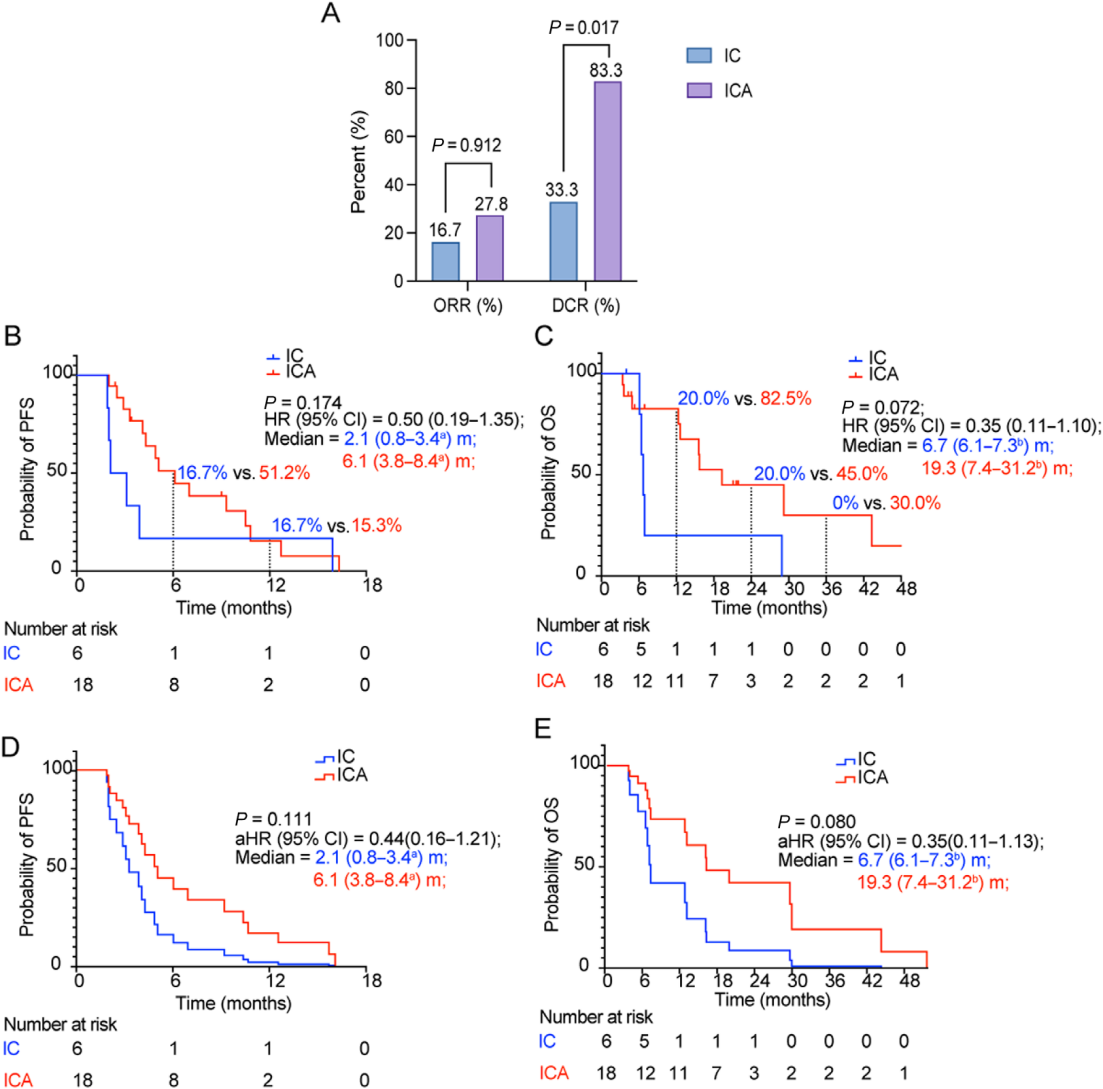
Treatment outcomes for brain metastasis patients (*n* = 24) by immunotherapy regimens. (A) ORR and DCR for IC group and ICA group in brain metastasis patients; (B) and (C) PFS and OS for IC group and ICA group in brain metastasis patients by Kaplan–Meier method with log‐rank test; (D) and (E) PFS and OS for IC group and ICA group in brain metastasis patients by Cox proportional hazards models. IC, PD‐1 inhibitors combined with chemotherapy; ICA, PD‐1 inhibitors combined with chemotherapy plus anti‐VEGFR agents; ORR, objective response rate; DCR, disease control rate; PFS, progression‐free survival; OS, overall survival; HR, hazard ratio; aHR, adjust hazard ratio; CI, confidence interval; *Note*: ^a^95% CI of median PFS; ^b^95% CI of median OS.

Among the 24 patients with brain metastasis included in this study, patients receiving antiangiogenic‐containing triple therapy (ICA) demonstrated significantly superior disease control versus IC therapy, evidenced by a 50.0% absolute improvement in DCR (83.3 vs. 33.3%, aOR 23.75, 95% CI 1.78–317.05, *p *= 0.017, posthoc power = 55.4%). Numerically longer median PFS (6.1 vs. 2.1 months, aHR 0.44, 95% CI 0.16–1.21; *p *= 0.111) and median OS by 12.6 months (19.3 vs. 6.7 months, aHR 0.35, 95% CI 0.11–1.13; *p *= 0.080) were observed in the ICA group, though these differences did not reach statistical significance. A numerical increase in ORR was also noted (27.8 vs. 16.7%, Δ+11.1%, aOR 1.13, 95% CI 0.14–9.00, *p *= 0.912) (Figure 4). Collectively, these findings suggest that a more intensive combination strategy incorporating antiangiogenic agents with immunotherapy (ICA) may be considered for patients with TNBC and brain metastases in clinical practice.

#### 2.3.2 Therapeutic Intensification in Early‐Recurrence (DFI < 12 Months) TNBC

Patients exhibiting early disease recurrence (disease‐free interval [DFI] < 12 months, *n* = 54) demonstrated significantly inferior clinical outcomes compared with those with DFI ≥ 12 months, including reduced DCR (64.8 vs. 90.1%, aOR 5.36, 95% CI 2.26–12.68, *p *< 0.001), shorter median PFS (4.4 vs. 9.5 months, aHR 0.57, 95% CI 0.39–0.84; *p *= 0.005), and decreased median OS (13.3 vs. 28.9 months, aHR 0.53, 95% CI 0.33–0.86; *p *= 0.009) (Figure S3).

Current evidence indicates that early disease relapse correlates with diminished clinical benefit from immunotherapy in TNBC. This prompted us to investigate whether the addition of antiangiogenic therapy could enhance treatment efficacy in this high‐risk population. Notably, among patients with early relapse, incorporating antiangiogenic agents into IC (ICA group) resulted in a 28.6% absolute improvement in DCR (78.6 vs. 50.0%, aOR 4.25, 95% CI 1.21–14.93, *p *= 0.024) and a 19.5% numerical increase in ORR (46.4 vs. 26.9%, aOR 2.45, 95% CI 0.76–7.89, *p *= 0.133) compared with IC alone (IC group), along with improved long‐term survival rates (Table 3). Collectively, these findings suggest that patients with highly aggressive TNBC may require a more intensive combination immune regimen. Therefore, for patients with early recurrence or metastasis (DFI < 12 months), a more aggressive treatment approach incorporating immunotherapy and antiangiogenic agents may be warranted.

**TABLE 3:** Efficacy outcomes in early recurrence patients.

	DFI < 12 months *N* = 54		
	IC group	ICA group	*p* Value
*N* (%)	26 (48.1)	28 (51.9)	
ORR (%)	7 (26.9)	13 (46.4)	0.133
95% CI	8.7–45.2	26.7–66.1	
DCR (%)	13 (50.0)	22 (78.6)	0.024
95% CI	29.4–70.6	62.4–94.8	
**Therapy line**			
Line 1–2	23 (88.5)	22 (78.6)	0.470
Line ≥ 3	3 (11.5)	6 (21.4)	
PFS (median)	3.5m	4.5m	0.646
95% CI	1.4–5.7	3.2–5.8	
≥6m	8 (44.3)	7 (31.8)	
≥1y	2 (13.9)	5 (27.2)	
≥2y	1 (6.9)	1 (9.1)	
OS (median)	11.0m	15.6m	0.708
95% CI	8.0–14.0	8.0–23.2	
≥1y	7 (49.7)	13 (62.6)	
≥2y	1 (12.4)	3 (28.3)	
≥3y	1 (12.4)	2 (18.8)	

Abbreviations: CI, confidence interval; DCR, disease control rate; IC group, PD‐1 inhibitors combined with chemotherapy; ICA group, PD‐1 inhibitors combined with chemotherapy plus anti‐VEGFR agents; ORR, objective response rate; OS, overall survival; PFS, progression‐free survival.

## 3 Discussion

Increasing clinical evidence supports a role for immunotherapy in TNBC. While these advances have reshaped clinical practice, significant challenges persist. Real‐world data are essential to inform therapeutic decision‐making. This retrospective study compared IC versus ICA therapy in advanced TNBC. Despite a higher baseline disease burden in the ICA group (more late‐line and brain metastasis patients), ICA achieved a significantly higher DCR and numerical improvements in 1‐, 2‐, and 3‐year OS, suggesting added benefit trend with anti‐VEGFR therapy. Earlier‐line immunotherapy was associated with better outcomes, and ICA suggested potential benefits in the heavily pretreated (≥third line) setting.

Although IMpassion130 and KEYNOTE‐355 established the first‐line IC for PD‐L1‐positive advanced TNBC [15, 16], and TORCHLIGHT confirmed toripalimab's efficacy [26], approximately 60% of TNBC patients are PD‐L1‐negative and derive limited benefit from immunotherapy, highlighting a critical unmet medical need [15, 16, 17]. Chemotherapy remains standard in PD‐L1‐negative patients, necessitating novel strategies [27, 28]. Notably, our study observed no significant differences in ORR, DCR, PFS, or OS between the CPS ≥ 10 vs. <10 subgroups, potentially mediated by anti‐VEGFR modulation. This suggests that the role of PD‐L1 CPS as a standalone predictive biomarker may be confounded by factors such as assay variability (SP142 vs. 22C3), sample heterogeneity, and arbitrary cutoffs [29, 30, 31, 32, 33].

Preclinical evidence indicates antiangiogenic agents reverse VEGF‐mediated immunosuppression, promote T‐cell infiltration [34], induce vascular normalization [35], and synergize with PD‐1/PD‐L1 blockade [22]. The FUTURE‐C‐PLUS trial [36] and several clinical studies [22, 23, 24] further supports that combining antiangiogenesis with immunotherapy may overcome PD‐L1 expression limitations. However, whether PD‐L1‐positive patients derive additional benefit from combined antiangiogenic therapy remains unclear. Our results from exploratory analysis revealed that among patients in the CPS < 1 subgroup, even though 55.6% were receiving third‐line or later treatment, the addition of antiangiogenic agents achieved an ORR comparable to that of patients with CPS ≥10. Therefore, it may be reasonable to actively consider adding antiangiogenic agents to immunotherapy regimens for patients with CPS < 1. However, these findings are purely descriptive (no statistical testing was performed due to the very small sample size) and require validation in larger prospective studies.

Brain metastases occur in 26–46% of advanced TNBC patients and face an extremely poor prognosis (median OS approximately 6 months) [37, 38, 39]. Overcoming the blood–brain barrier remains a key challenge [39, 40, 41]. Preclinical and clinical evidence suggests that anti‐VEGFR agents may enhance blood–brain barrier penetrability. Notably, a 2025 ASCO presentation reported that the combination of ICA therapy achieved a remarkable central nervous system ORR of 77.1% in TNBC patients with brain metastasis [42]. In our analysis, adding anti‐VEGFR to IC (ICA) significantly improved DCR by 50.0% (*p* = 0.017), with numerical extensions in PFS (+4.0 months) and OS (+12.6 months) that did not reach statistical significance. Collectively, these findings are consistent with the significant central nervous system ORR data reported at ASCO 2025 and suggest that incorporating antiangiogenic therapy for TNBC brain metastases may provide greater benefit although the survival trends did not reach statistical significance. Therefore, larger prospective studies are warranted for confirmation.

TNBC patients experiencing early recurrence (DFI < 12 months) present highly aggressive tumors characterized by primary chemotherapy resistance and poor prognosis [43, 44, 45, 46], creating an urgent need for more effective therapies. The IMpassion132 trial specifically enrolled this population but found no significant OS benefit with atezolizumab plus chemotherapy versus chemotherapy alone in PD‐L1‐positive patients [24]. Similarly, KEYNOTE‐355 subgroup analysis showed no added efficacy for PD‐1 blockade in patients recurring within 6–12 months, collectively indicating limited immunotherapy benefit in early‐recurrence TNBC. Prompted by this unmet need, we investigated whether adding antiangiogenic agents could enhance immunotherapy efficacy [16]. In the early‐recurrence cohort, triple therapy significantly improved DCR (*p* = 0.024) and achieved numerically higher ORR (*p* = 0.133). A trend toward improved long‐term survival was also observed, but these differences were not statistically significant. These results support intensifying therapy with antiangiogenic combinations in this high‐risk population and warrant clinical consideration.

A major limitation of this study is the absence of comprehensive adverse event (AE) data, which precludes direct assessment of the toxicity profile of the ICA regimen. Nevertheless, expected toxicities can be informed by published prospective studies. In a triplet combination study [25], camrelizumab, apatinib, and eribulin were associated with Grade 3–4 AEs in 48% of patients. The most frequent events were neutropenia (28%) and hypertension (16%). No treatment related deaths occurred. Common all‑grade AEs included fatigue, hand–foot syndrome, proteinuria, and immune‑related events such as rash and hypothyroidism. Similarly, the ATRACTIB trial [24] noted hypertension, proteinuria, and diarrhea as frequent bevacizumab‑related AEs. When combining anti‑VEGF agents with ICIs and chemotherapy, clinicians should monitor for overlapping toxicities, notably hypertension, bleeding, and immune‑mediated events. Our retrospective data cannot quantify these risks, underscoring the need for prospective safety evaluation.

This study has several other limitations inherent to its retrospective design. First, the sample size, particularly in exploratory subgroup analyses (e.g., brain metastasis, CPS < 1), was modest, limiting statistical power and the ability to detect significant differences for some outcome trends. Second, PD‐L1 biomarker data were incomplete for a portion of the cohort, which may introduce selection bias and affect the generalizability of biomarker‐related findings. Third, baseline imbalances exist between groups. Notably, the ICA group included more patients with ≥3 prior lines of therapy and brain metastases. These imbalances may introduce residual confounding, even after multivariable adjustment. Unmeasured factors such as performance status, prior treatment response, and tumor genomic features could influence outcomes. The observed benefit of ICA, despite a higher baseline disease burden, suggests potential efficacy. Nevertheless, the nonrandomized design precludes definitive causal attribution. Therefore, subgroup findings should be interpreted with caution, and prospective validation is required. Additionally, response assessments were based on real‑world clinical imaging and may not be as standardized as those in clinical trials, which could affect the accuracy of the DCR evaluation. Finally, despite statistical adjustments, unmeasured confounding factors may persist. These limitations underscore the need for cautious interpretation of the results and highlight the necessity of prospective, randomized controlled trials for validation.

In conclusion, this real‐world study suggests that adding antiangiogenic agents to standard immunochemotherapy enhances disease control in advanced TNBC. Statistically significant improvements in DCR were observed in the overall population and in patients with ≥3 prior lines of therapy, brain metastases, and early recurrence. Nonsignificant numerical trends were observed for ORR, PFS, and OS in several subgroups. Descriptive findings without statistical testing are reported for the CPS < 1 subgroup. The benefits appeared independent of PD‐L1 status, challenging its sole predictive role and proposing ICA as a potential strategy to overcome PD‐L1‐negative resistance. These results require cautious interpretation, and future research should focus on validating these results in randomized trials and comprehensively evaluating the efficacy‐safety balance of intensified combination regimens in advanced TNBC.

## 4 Methods

### 4.1 Study Design and Participants

This retrospective cohort study enrolled 271 patients with histologically confirmed advanced TNBC (ER < 1%, HER2‐negative) treated with PD‐1 inhibitors at Sun Yat‐sen Memorial Hospital Breast Cancer Center from January 2019 to December 2024. Its inclusion criteria comprised: (1) histologically confirmed advanced TNBC (ER < 1% by IHC; HER2‐negative per ASCO/CAP guidelines); (2) measurable disease (RECIST v1.1); (3) ≥1 cycle of immunotherapy–chemotherapy ± anti‐VEGFR agents; (4) complete baseline and follow‐up data. Exclusion criteria included: ER ≥ 1% or HER2 positivity (*n* = 28), lack of follow‐up data (<1 cycle of treatment or loss to follow‐up before first radiographic evaluation) (*n* = 25).

Baseline characteristics extracted from electronic medical records included: age, histological type, visceral/brain metastasis, DFI (defined as the time from completion of curative‐intent (neo)adjuvant therapy to the first recurrence or metastasis for non‐de novo Stage IV patients), treatment line of therapy (line 1–2 vs. line ≥ 3), specific immunotherapy/anti‐VEGFR agents, PD‐L1 CPS (using 22C3 pharmDx assay; CPS ≥ 10 cutoffs).

*Treatment Regimens*: Immunotherapy involved PD‐1 inhibitors (pembrolizumab 200 mg iv q3w, camrelizumab 200 mg iv q3w, or toripalimab 240 mg iv q3w). Chemotherapy drugs included nab‐paclitaxel (100–125 mg/m^2^ iv on days 1, 8, 15 q4w), gemcitabine (800–1000 mg/m^2^ iv on days 1, 8 q3w), platinum agents (carboplatin AUC 5 or cisplatin 75 mg/m^2^ iv q3w), or eribulin (1.4 mg/m^2^ iv on days 1, 8 q3w). Anti‐VEGFR agents included apatinib (250 mg po qd) or bevacizumab (7.5 mg/kg iv q3w). The choice of agent, dose, and specific combination was at the treating physician's discretion based on standard practice and patient tolerance.

Efficacy objective was assessed by Response Evaluation Criteria in Solid Tumors (RECIST) 1.1: (1) ORR, complete/partial response (CR+PR), (2) DCR, CR+PR+stable disease (SD), (3) PFS/OS, defined as the time from immunotherapy initiation to progression/death or last follow‐up (February 2025).

### 4.2 Statistical Analysis

Statistical analyses were conducted using SPSS 27.0 for Windows with the following prespecified approach. Missing data within the included data were assumed to be missing at random. All statistical tests were two‐tailed, and significance was defined as a *p* value <0.05. Categorical variables were compared using Fisher's exact test. Multivariable logistic regression models were employed to estimate aORs with 95% CIs for ORR and DCR, adjusting for prespecified covariates including line of therapy (1–2 vs. ≥3) and brain metastasis (yes vs. no). Survival curves were generated by Kaplan–Meier method with log‐rank test. Multivariable Cox proportional hazards models were employed to estimate aHRs with 95% CIs for PFS and OS, adjusting for prespecified covariates including line of therapy (1–2 vs. ≥3) and brain metastasis (yes vs. no). Subgroup analyses prespecified for: (1) PD‐L1 CPS stratification (CPS < 10/≥10), (2) high‐risk populations: brain metastasis, early relapse (DFI < 12 months). For exploratory subgroup analyses (brain metastases, CPS < 1, and ≥3 prior lines), posthoc power calculations were performed using two‐sample proportion tests (for DCR/ORR) and log‐rank tests (for PFS/OS) with an alpha level of 0.05. The observed effect sizes (e.g., DCR difference of 50.0% in brain metastasis cohort) and actual sample sizes were used to estimate statistical power.

## Author Contributions

Concept and design: Qiang Liu, Yudong Li, and Jinna Lin. Acquisition, analysis, or interpretation of data: all authors. Drafting of the manuscript: Yudong Li, Jinna Lin, and Mengdi Zhu. Critical revision of the manuscript: all authors. Statistical analysis: Yudong Li and Jinna Lin. Obtained funding: Qiang Liu. All authors have read and approved the final manuscript.

## Funding Information

This work was supported by the National Natural Science Foundation of China (82061148016, 82230057, 82272859, 82203087), National Key Research and Development Program of China (2022YFC2505101), Guangdong Provincial Clinical Research Center for Breast Diseases (2023B110005), and National Science and Technology Major Project (2025ZD0552502).

## Ethics Statement

The study protocol received approval from the Institutional Review Board of Sun Yat‐sen Memorial Hospital (SYSKY‐2025‐663‐01), with waiver of informed consent for retrospective anonymized data.

## Conflicts of Interest

The authors declare no conflicts of interest.

## Supporting information




**Figure S1** Survival outcomes by IC and ICA group (A) and (B) PFS and OS of IC and ICA group by Kaplan–Meier method with log‐rank test; (C) and (D) PFS and OS of IC and ICA group by Cox proportional hazards models. *Abbreviations*: PFS, progression‐free survival; OS, overall survival; HR, hazard ratio; aHR, adjust hazard ratio; CI, confidence interval; Note: ^a^95% CI of median PFS; ^b^95% CI of median OS.
**Figure S2** Treatment response and survival outcomes by therapy line. (A) ORR and DCR of different therapy line; (B) and (C) PFS and OS of different therapy line by Kaplan–Meier method with log‐rank test; (D) and (E) PFS and OS of different therapy line by Cox proportional hazards models. *Abbreviations*: ORR, objective response rate; DCR, disease control rate; PFS, progression‐free survival; OS, overall survival; HR, hazard ratio; aHR, adjust hazard ratio; CI, confidence interval; Note: ^a^95% CI of median PFS; ^b^95% CI of median OS.
**Figure S3** Treatment outcomes of different DFI patients. (A) ORR and DCR for different DFI patients; (B) and (C) PFS and OS for different DFI patients by Kaplan–Meier method with log‐rank test; (D) and (E) PFS and OS for different DFI patients by Cox proportional hazards models. *Abbreviations*: DFI, disease‐free interval; ORR, objective response rate; DCR, disease control rate; PFS, progression‐free survival; OS, overall survival; HR, hazard ratio; CI, confidence interval; Note: ^a^95% CI of median PFS; ^b^95% CI of median OS.
**Table S1** Efficacy of immunotherapy combination regimens stratified by PD‐L1 CPS status.

## Data Availability

The data presented in this study are available on request from the corresponding author. The data are not publicly available due to privacy issue.
