# Supplementary material for: Chemoimmunotherapy Combined With Antiangiogenic Therapy in Advanced Triple‐Negative Breast Cancer: Real‐World Outcomes From a Retrospective Cohort Study
Source: MedComm (2020). 2026 Jul 5;7(7):e70839. doi: 10.1002/mco2.70839 (PMC13334144; doi:10.1002/mco2.70839)
Supplement: Supplementary file 1 — Figure S1 Survival outcomes by IC and ICA group (A) and (B) PFS and OS of IC and ICA group by Kaplan–Meier method with log‐rank test; (C) and (D) PFS and OS of IC and ICA group by Cox proportional hazards models. Abbreviations: PFS, progression‐free survival; OS, overall survival; HR, hazard ratio; aHR, adjust hazard ratio; CI, confidence interval; Note: a95% CI of median PFS; b95% CI of median OS. Figure S2 Treatment response and survival outcomes by therapy line. (A) ORR and DCR of different therapy line; (B) and (C) PFS and OS of different therapy line by Kaplan–Meier method with log‐rank test; (D) and (E) PFS and OS of different therapy line by Cox proportional hazards models. Abbreviations: ORR, objective response rate; DCR, disease control rate; PFS, progression‐free survival; OS, overall survival; HR, hazard ratio; aHR, adjust hazard ratio; CI, confidence interval; Note: a95% CI of median PFS; b95% CI of median OS. Figure S3 Treatment outcomes of different DFI patients. (A) ORR and DCR for different DFI patients; (B) and (C) PFS and OS for different DFI patients by Kaplan–Meier method with log‐rank test; (D) and (E) PFS and OS for different DFI patients by Cox proportional hazards models. Abbreviations: DFI, disease‐free interval; ORR, objective response rate; DCR, disease control rate; PFS, progression‐free survival; OS, overall survival; HR, hazard ratio; CI, confidence interval; Note: a95% CI of median PFS; b95% CI of median OS. Table S1 Efficacy of immunotherapy combination regimens stratified by PD‐L1 CPS status. [file MCO2-7-e70839-s001.pdf]

Chemoimmunotherapy Combined with Anti-Angiogenic Therapy in Advanced Triple-Negative Breast Cancer: Real-World Outcomes from a Retrospective Cohort Study

Yudong Li[1,2#], Jinna Lin[1,2#], Mengdi Zhu[1,2#], Zijie Cai[1,2], Liang Jin[1,2], Shunying Li[1,2], Qianfeng Shi[1,2], Hongna Lai[1,2], Chang Gong[1,2], Qiang Liu[1,2*]

[1]Guangdong Provincial Key Laboratory of Malignant Tumor Epigenetics and Gene Regulation, Sun Yat-Sen Memorial Hospital, Sun Yat-Sen University, 510120, Guangzhou, China

[2]Breast Tumor Center, Sun Yat-Sen Memorial Hospital, Sun Yat-Sen University, 510120, Guangzhou, China

#Contributed Equally

*Corresponding Author

Corresponding Author

Qiang Liu, Breast Tumor Center, Sun Yat-Sen Memorial Hospital, Sun Yat-Sen University, 510120, Guangzhou, Guangdong, China (liuq77@mail.sysu.edu.cn).

Supporting Information for review and online publication only

Supplementary Figures

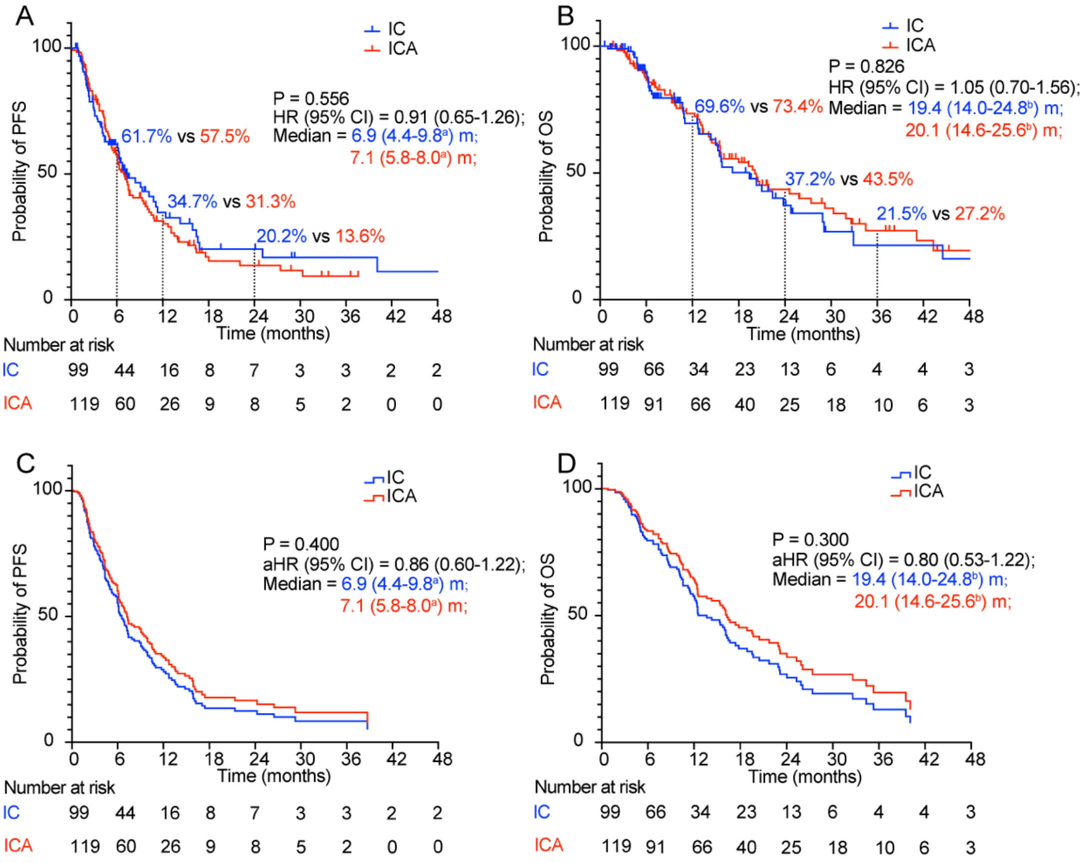

Figure S1 Survival Outcomes by IC and ICA group

A) and B) PFS and OS of IC and ICA group by Kaplan-Meier method with log-rank test; C) and D) PFS and OS of IC and ICA group by Cox proportional hazards models. Abbreviations: PFS, Progression-free survival; OS, Overall survival; HR, hazard ratio; aHR, adjust hazard ratio; CI, confidence interval; Note: a95% CI of median PFS; b95% CI of median OS;

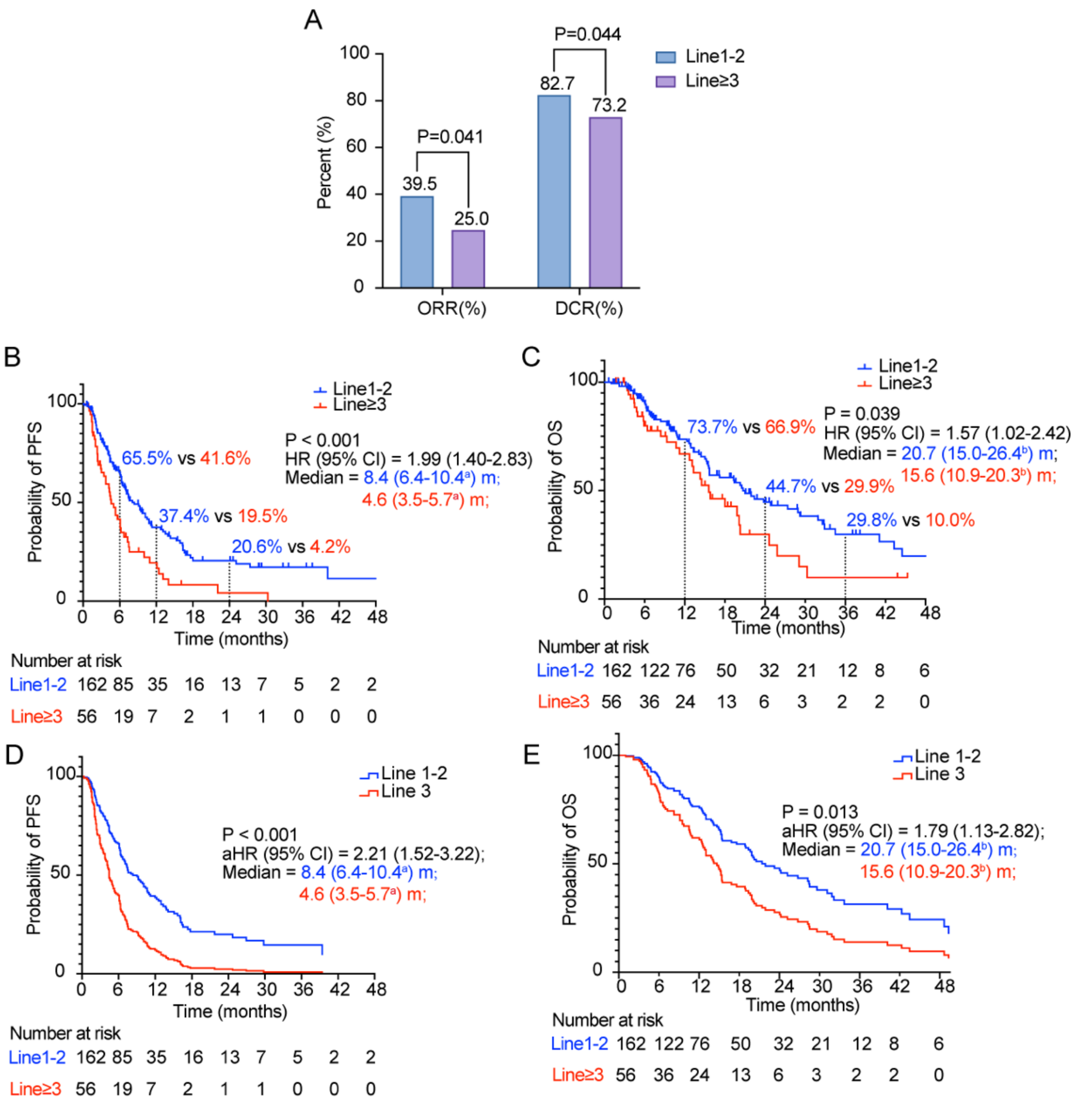

Figure S2. Treatment Response and Survival Outcomes by Therapy Line.

A) ORR and DCR of different therapy line; B) and C) PFS and OS of different therapy line by Kaplan-Meier method with log-rank test; D) and E) PFS and OS of different therapy line by Cox proportional hazards models. Abbreviations: ORR, Objective response rate; DCR, disease control rate; PFS, Progression-free survival; OS, Overall survival; HR, hazard ratio; aHR, adjust hazard ratio; CI, confidence interval; Note: [a]95% CI of median PFS; [b]95% CI of median OS;

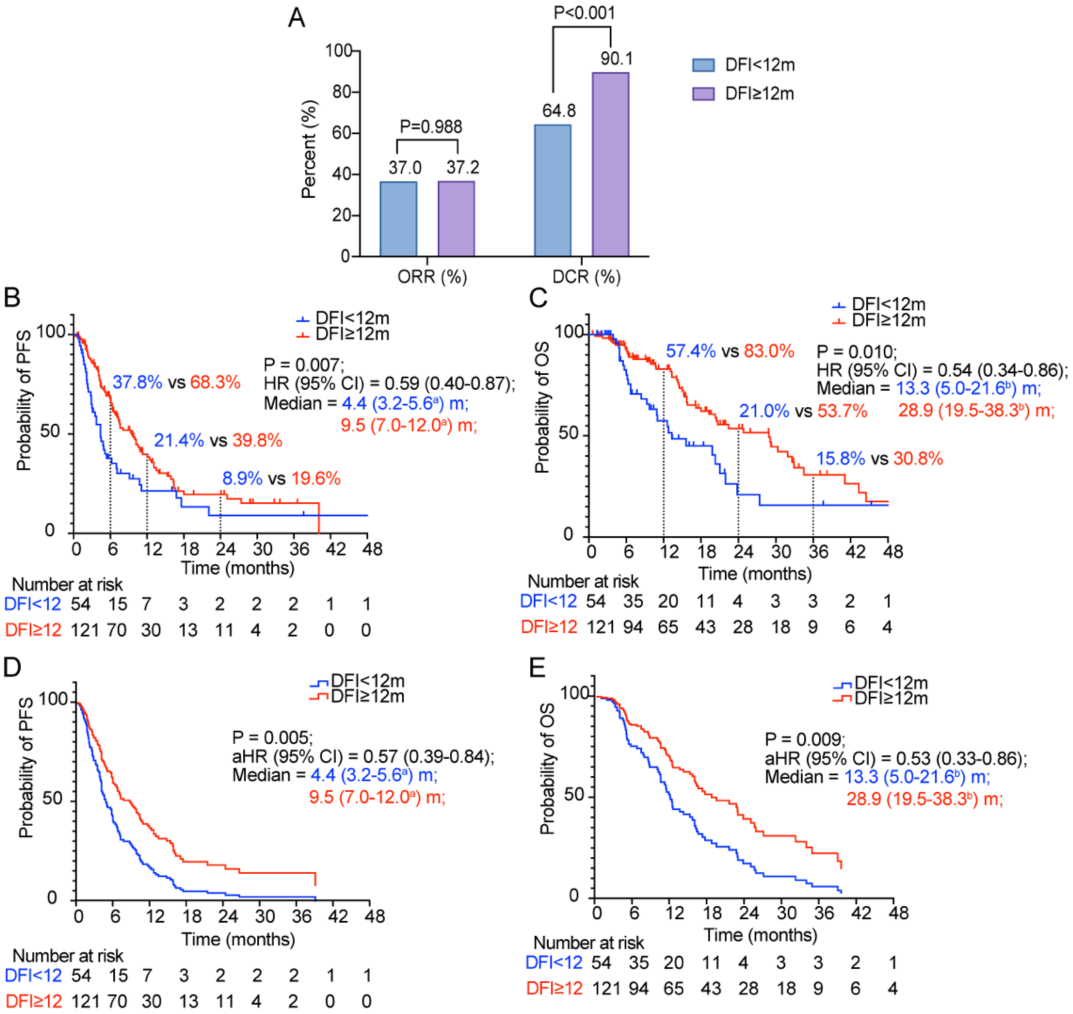

Figure S3. Treatment outcomes of different DFI patients.

A) ORR and DCR for different DFI patients; B) and C) PFS and OS for different DFI patients by Kaplan-Meier method with log-rank test; D) and E) PFS and OS for different DFI patients by Cox proportional hazards models. Abbreviations: DFI, disease-free interval; ORR, Objective response rate; DCR, disease control rate; PFS, Progression-free survival; OS, Overall survival; HR, hazard ratio; CI, confidence interval; Note: [a]95% CI of median PFS; [b]95% CI of median OS;

Supplementary Tables

Supplemental Table 1 Efficacy of Immunotherapy Combination Regimens Stratified by PD-L1 CPS Status

	CPS ≥10 N=55		*P*-value	CPS<10 N=68		*P*-value	CPS<1
	IC group	ICA group		IC group	ICA group		ICA group
**N (%)**	28 (50.9)	27 (49.1)		28 (41.2)	40 (58.8)		18 (62.1)
**ORR (%)**	11 (39.3)	10 (37.0)	0.864	7 (25.0)	15 (37.5)	0.278	7 (38.9)
**95% CI**	20.0-58.6	17.6-56.5		7.9-42.1	21.8-53.2		13.9-63.8
**DCR (%)**	19 (67.9)	21 (77.8)	0.409	22 (78.6)	34 (85.0)	0.494	16 (88.9)
**95% CI**	49.4-86.3	61.0-94.5		62.4-94.8	73.4-96.6		72.8-100
**Therapy line**							
line1-2	22 (78.6)	18 (66.1)	0.464	20 (71.4)	25 (62.5)	0.731	8 (44.4)
line≥3	6 (21.4)	9 (33.9)		8 (28.6)	15 (37.5)		10 (55.6)
**PFS (median)**	7.1m	6.3m	0.732	6.7m	5.9m	0.850	7.5m
**95% CI**	2.8-11.4	3.5-9.1		3.6-9.8	3.2-8.6		4.0-11.0
≥6m	13 (58.1)	12 (57.6)		13 (67.7)	17 (49.1)		8 (50.9)
≥1y	5 (33.7)	6 (38.4)		5 (41.3)	10 (38.6)		5 (43.6)
≥2y	4 (33.7)	2 (21.3)		1 (15.5)	5 (19.3)		2 (17.4)
**OS (median)**	20.3m	19.8m	0.774	15.8m	15.6m	0.736	20.1m
**95% CI**	12.2-28.4	16.9-22.7		11.2-20.4	5.1-26.1		7.1-33.1
≥1y	10 (64.2)	16 (81.7)		10 (78.6)	16 (57.5)		10 (77.4)
≥2y	5 (36.7)	5 (42.5)		3 (35.9)	10 (42.6)		6 (46.4)
≥3y	1 (27.5)	3 (34.0)		1 (18.0)	5 (33.1)		3 (31.0)

Abbreviations: IC group = PD-1 inhibitors combined with chemotherapy; ICA group = PD-1 inhibitors combined with chemotherapy plus anti-VEGFR agents; ORR, Objective response rate; DCR, disease control rate; CI, confidence interval; PFS, Progression-free survival; OS, Overall survival;

The authors have provided this supporting information solely for review and online publication, offering readers additional details about their work.
